# Digital Eye Strain among Adults Presenting to Tertiary Care Hospital in the Era of COVID-19 Pandemic: A Descriptive Cross-sectional Study

**DOI:** 10.31729/jnma.7092

**Published:** 2022-01-31

**Authors:** Anjila Basnet, Samyam Bickram Pathak, Anurag Marasini, Rohit Pandit, Amita Pradhan

**Affiliations:** 1 Department of Ophthalmology, KIST Medical College and Teaching Hospital, Lalitpur, Nepal; 2 Community Dentistry, People's Dental College and Hospital, Kathmandu, Nepal

**Keywords:** *COVID-19*, *eyestrain*, *pandemic*

## Abstract

**Introduction:**

The Novel Coronavirus disease 2019 pandemic has sent humanity indoors, replacing human contact with an electronic connection. The mandatory online classes and work from home policy to maintain the social distancing during the pandemic has forced the individual to spend most of the time in front of laptops or mobile screens. Digital eye strain is a group of vision-related symptoms that result from the continuous use of devices with digital displays, such as computers, tablets, and smartphones. The present study is done to find out the prevalence of digital eye strain among the adult population in a tertiary care hospital in the era of the COVID-19 pandemic.

**Methods:**

This descriptive cross-sectional study was done from 16^th^ January 2021 to 31^st^ July 2021 in a tertiary care hospital of Nepal after receiving ethical approval from the Institutional Review Committee (Registration number: 077/78/30). Convenience sampling was done. The sample size calculated in our study was 322. Data collection and entry were done in Microsoft Excel, point estimate at 95% Confidence Interval was calculated along with frequency and proportion for binary data.

**Results:**

The prevalence of digital eye strain was 300 (94.3%) (91.8-96.8 at 95% Confidence Interval) among 318 respondents. Eye strain (irritation, heaviness) was the most common digital eye strain symptom 199 (62.6%) followed by the tiredness of eyes 162 (50.9%).

**Conclusions:**

The present study concluded that the prevalence of digital eye strain in the era of COVID-19 is high as compared to other studies conducted among adults.

## INTRODUCTION

The mandatory online classes and work from home policy to maintain the social distancing during the Coronavirus disease 2019 pandemic has forced the individual to spend most of the time in front of laptops or mobile screens. Without any specific guidelines, it is now a usual routine for individuals to spend many hours on a digital screen. This will mostly lead to several symptoms related to vision and probably musculoskeletal issues.^1^ prolonged use of a computer screen and other visual display terminals can lead to digital eye strain.

The American Optometric Association defines Digital Eye Strain as the complex of eye and vision problems related to near work, which may be experienced during or related to computer use. Many individuals experience eye discomfort with the use of Visual Display Terminals.^2^

The study aimed to find out the prevalence of digital eye strain among adults in a tertiary care hospital in the era of the COVID-19 pandemic.

## METHODS

This descriptive cross-sectional study was conducted in KIST Medical College and Teaching Hospital, Imadol, Lalitpur, from 16^th^ January 2021 to 31^st^ July 2021. Ethical clearance was taken from the institutional review board of KIST Medical College and Teaching Hospital with registration number 077/78/30. The study population was the participants attending Ophthalmology Outpatient Department of a tertiary hospital. The inclusion criteria for the study were patients (>20 years) and using the digital screen daily for one hour or more over some months/years. Participants who did not give consent for the study and were unable to provide the required information or submitted an incomplete questionnaire form were excluded from the study.

Data collection was done by filling a semi-structured research questionnaire which was prepared after reviewing the articles available on computer vision syndrome.^3^ Data were collected in demography details, spectacles use, digital screen use, selfreported symptoms of digital eye strain, any measures practiced to prevent eye problems, radiation filter. Data was collected throughout the study period to meet the sample size for the study.

Convenience sampling was done and the sample size was calculated using the formula given below:

n = Z^2^ × (p × q) / e^2^

  = (1.96) ^2^ × 0.5 × (1-0.5) / (0.06)^2^

  = 267

Where,

n= required sample size
Z= 1.96 at 95% Confidence Interval (CI)
p= prevalence of digital eye strain among the adult population in a tertiary care hospital in the era of the COVID-19 pandemic taken as 50% for maximum sample size
e= margin of error, 6% in this study

Hence, the sample size for the study was calculated and found to be 267. After addressing 10% for nonresponse, the final sample size calculated was 297. However, a sample size of 322 was taken.

Selection and information bias has been minimized as possible. Data entry was done in Microsoft Excel, point estimate at 95% CI was calculated along with frequency and proportion for binary data, and the analysis was done.

## RESULTS

A total of 322 participants were recruited for the study. Out of them, four were subsequently excluded because of incomplete data. The final response rate was 318 (98.7%), out of which 145 (45.6%) were male and 173 (54.4%) were female. The prevalence of symptoms of digital eye strain (one or more) was 300 (94.3%) (91.8-96.8% at 95% Confidence Interval) among 318 respondents. The mean age of study participants was 36 years. Eye strain (irritation, heaviness) was the most common digital eye strain symptom 199 (62.6%) followed by the tiredness of eyes 162 (50.9) (Table 1).

**Table 1:** Prevalence of symptoms among participants presenting with digital eye strain (n= 318).

Symptoms	n (%)
Eye strain (irritation, heaviness)	199 (62.6)
Tiredness of eyes	162 (50.9)
Headache	140 (44)
Discomfort	101 (31.8)
Watering of eyes	89 (28)
Blurring of vision	81 (25.5)
Dry eye	64 (20.1)
Redness of eyes	46 (14.5)
Backache	44 (13.8)
Neck pain	44 (13.8)
Shoulder pain	40 (12.6)
Double vision	18 (5.7)

Out of 318 participants, 152 (47.8%) were wearing spectacles. The majority of them were myopes 110 (34.6%) followed by astigmatism 25 (7.9%) and hypermetropia 17 (5.3%). Among the respondents, 312 (98.1%) were aware that prolonged use of digital screens has bad effects on the eyes. The majority 108 (34%) of participants spent 2-4 hours on the digital screen and 192 (60.4%) experienced the symptoms of digital eye strain after 1-2 hours of digital screen use. The duration spent by the respondents on the computer (Table 2).

**Table 2:** Average hours spent on the computer daily (n= 318).

Hours per day	n (%)
1-2	93 (29.2)
2-4	108 (34)
4-6	72 (22.6)
6-8	24 (7.5)
8-10	14 (4.4)
>10	7 (2.2)
Total	318 (100)

Taking breaks in between the use of a computer, 244 (76.7%) was the most common preventive measure taken for relief of symptoms of digital eye strain. While working on the computer, 187 (58.8%) participants used fluorescent light in the room, and 169 (53.1%) kept the computer screen at eye level.

## DISCUSSION

One of the most important ways to reduce the potential spread of COVID-19 is to remain at home and minimize physical proximity. This, however, has led to increased use of digital devices and screen time for both work and recreation, which may lead to an increase in eye strain or other ocular complications. A study done by Sitaula, et al. revealed that the prevalence of digital eye strain was significantly high.^4^ Digital eye strain is a group of visual symptoms experienced concerning the use of computers. Nearly 60 million people suffer from DES globally, resulting in reduced productivity at work and reduced quality of life of computer workers.^5^ The prevalence of symptoms of DES (one or more) was found out to be 94.3% in our study. The finding was similar to the study done in Malaysia in which the prevalence of symptoms of DES was found to be 89.9%.^3^ The incidence of DES among computer users is reported to be between 64% and 90%.^6^ A study done by Dessie, et al. concluded with a prevalence of DES as 69.5%.^7^ Likewise in a study by Tauste, et al. the overall prevalence of DES was 53%.^8^ Another study reviewed by Sheppard, et al. concluded that the prevalence of DES maybe 50% or more among computer users indicating that a large proportion of the population is at risk, and may seek advice or treatment linked to the condition.^9^

This study described the commonest reported symptoms of DES as eye strain (62.6%) followed by the tiredness of eyes (50.9%). This finding was strongly supported by the study done by Bali, et al. in which the chief presenting symptoms were eyestrain (97.8%), tiredness (79.1%), and redness (61.2%).^10^ Meanwhile, one study showed the common symptoms were tired eyes and headache.^11^ A study reviewed by Rosenfield concluded that the prevalence of symptoms including eyestrain, headache, ocular discomfort, dry eye, diplopia, and blurred vision may be as high as 90%.^12^ In a study done by Poudel S, et al. DES symptoms included headache (48%), tired eyes (47%), and eye strain (43%).^13^

The limitation of this study was that the temporal link between outcome and exposure could not be determined because both were examined at the same time. It cannot be used to establish cause and effect relationships as respondents may not be truthful while answering survey questions. The choice and wording of questions on a questionnaire may even influence the descriptive findings.

## CONCLUSIONS

The present study concluded that the prevalence of digital eye strain in the era of COVID-19 is high as compared to other studies conducted among adults. Computers and other visual display devices have brought tremendous changes in overall lifestyle. The increased use of electronic devices and their influence on the wellbeing of users is a concern to healthcare practitioners. Digital eye strain is one of these health concerns. Serious attention is needed on this global problem.
